# Supplementary material for: Outcomes at 10-Year Follow-Up after Roux-en-Y Gastric Bypass, Biliopancreatic Diversion, and Sleeve Gastrectomy
Source: J Clin Med. 2023 Jul 28;12(15):4973. doi: 10.3390/jcm12154973 (PMC10419540; doi:10.3390/jcm12154973)
Supplement: Supplementary file 1 [file jcm-12-04973-s001.zip › jcm-2441950-supplementary.pdf]

Supplemental Table S1: Multivariate Regression Model - Percent of Total Weight Loss (%)

Predictor	Estimate	SE	Lower 95% CI	Upper 95% CI	t	p
Intercept [a]	24.2232	3.7700	16.81116	31.6353	6.425	< .001
Preop Weight	0.0323	0.0185	-0.00405	0.0686	1.747	0.081
Excess Weight (kg)	0.1638	0.0234	0.11780	0.2098	6.999	< .001
Age at appointment	-0.1589	0.0488	-0.25494	-0.0629	-3.254	0.001
Gender:						
F – M	3.0180	1.1553	0.74667	5.2893	2.612	0.009
Type of Operation:						
Lap RYGB with fundus excision – RYGB	-8.1436	2.1369	-12.34487	-3.9424	-3.811	< .001
BPD - LL – RYGB	0.5576	1.5819	-2.55253	3.6677	0.352	0.725
LAP – SG – RYGB	-2.0260	1.8794	-5.72099	1.6689	-1.078	0.282
Lap SG to RYGB conversion – RYGB	-2.1561	2.1529	-6.38884	2.0766	-1.002	0.317

[a] Represents reference level

Model Fit Measures

			Overall Model Test			
Model	R	R^2	F	df1	df2	p
1	0.482	0.233	14.8	8	389	< .001

Supplemental Table S2: Pairwise comparisons - Percentage of Excess Weight Loss

		**W**	**p**
RYGB	LAP RYGB LL with gastric fundus extraction	-5.113	**0.003**
RYGB	BPD - LL	2.501	0.392
RYGB	LAP – Sleeve Gastrectomy	-1.383	0.865
RYGB	LAP SG Redo LAP RYGB	-0.380	0.999
LAP RYGB LL with gastric fundus extraction	BPD - LL	5.245	**0.002**
LAP RYGB LL with gastric fundus extraction	LAP – Sleeve Gastrectomy	3.681	0.070
LAP RYGB LL with gastric fundus extraction	LAP SG Redo LAP RYGB	3.218	0.153
BPD - LL	LAP – Sleeve Gastrectomy	-2.830	0.266
BPD - LL	LAP SG Redo LAP RYGB	-1.503	0.826
LAP – Sleeve Gastrectomy	LAP SG Redo LAP RYGB	0.287	1.000

Supplemental Table S3: Multivariate Regression Model - BMI Difference

Predictor	Estimate	SE	t	p
Intercept [a]	-23.7379	4.0352	-5.8827	< .001
Excess Weight	-0.0470	0.0211	-2.2229	0.027
Preop Weight	0.0282	0.0171	1.6495	0.100
Preop BMI	0.8275	0.0610	13.5628	< .001
Gender:				
F – M	0.9818	1.0390	0.9449	0.345
Age at appointment	6.34e-4	0.0450	0.0141	0.989
Type of Operation (final):				
Lap RYGB with fundus excision – RYGB	-1.2598	1.9897	-0.6331	0.527
BPD - LL – RYGB	4.2383	1.4390	2.9454	0.003
LAP – SG – RYGB	6.6096	1.6401	4.0300	< .001
Lap SG to RYGB conversion – RYGB	3.1866	1.9465	1.6371	0.102

[a] Represents reference level

Supplemental Table S4: Multivariate Regression Model - Presence of Diabetes at Followup

Predictor	Estimate	SE	Z	p
Intercept	10.30632	2.95512	3.48761	< .001
Preoperative Diabetes:				
False – True	0.92471	0.72392	1.27736	0.201
Preop Weight	-8.18e−5	0.00973	-0.00841	0.993
Preop BMI	0.00822	0.03829	0.21473	0.830
Type of Operation (final):				
Lap RYGB with fundus excision – RYGB	-1.67779	0.82273	-2.03929	0.041
BPD - LL – RYGB	0.96464	1.09948	0.87737	0.380
LAP – SG – RYGB	16.42491	1498.57245	0.01096	0.991
Lap SG to RYGB conversion – RYGB	0.27264	1.10229	0.24734	0.805
Age at appointment	-0.15384	0.03604	-4.26909	< .001

Note. Estimates represent the log odds of "Presence of Diabetes at Follow up = False" vs. "Presence of Diabetes at Followup = True"
